# Strengthening capacity for AIDS vaccine research: analysis of the Pfizer Global Health Fellows Program and the International AIDS Vaccine Initiative

**DOI:** 10.1186/1472-6963-13-378

**Published:** 2013-10-02

**Authors:** Taryn Vian, Sayaka Koseki, Frank G Feeley, Jennifer Beard

**Affiliations:** 1 Center for Global Health and Development, Boston University, 801 Massachusetts Avenue, Boston, MA 02118, USA; 2 Department of International Health, Boston University School of Public Health, 801 Massachusetts Avenue, Crosstown Building 3rd floor, Boston, MA 02118, USA; 3 Abt Associates Inc., 4550 Montgomery Avenue, Suite 800N, Bethesda, MD 20814, USA

## Abstract

**Background:**

Industry partnerships can help leverage resources to advance HIV/AIDS vaccine research, service delivery, and policy advocacy goals. This often involves capacity building for international and local non-governmental organizations (NGOs). International volunteering is increasingly being used as a capacity building strategy, yet little is known about how corporate volunteers help to improve performance of NGOs in the fight against HIV/AIDS.

**Methods:**

This case study helps to extend our understanding by analyzing how the Pfizer Global Health Fellows (GHF) program helped develop capacity of the International AIDS Vaccine Initiative (IAVI), looking specifically at Fellowship activities in South Africa, Kenya, and Uganda. From 2005–2009, 8 Pfizer GHF worked with IAVI and local research centers to strengthen capacity to conduct and monitor vaccine trials to meet international standards and expand trial activities. Data collection for the case study included review of Fellow job descriptions, online journals, evaluation reports, and interviews with Fellows and IAVI staff. Qualitative methods were used to analyze factors which influenced the process and outcomes of capacity strengthening.

**Results:**

Fellows filled critical short-term expert staffing needs at IAVI as well as providing technical assistance and staff development activities. Capacity building included assistance in establishing operating procedures for the start-up period of research centers; training staff in Good Clinical Practice (GCP); developing monitoring capacity (staff and systems) to assure that centers are audit-ready at all times; and strategic planning for data management systems. Factors key to the success of volunteering partnerships included similarities in mission between the corporate and NGO partners, expertise and experience of Fellows, and attitudes of partner organization staff.

**Conclusion:**

By developing standard operating procedures, ensuring that monitoring and regulatory compliance systems were in place, training African investigators and community members, and engaging in other systems strengthening activities, the GHF program helped IAVI to accelerate vaccine development activities in the field, and to develop the organization’s capacity to manage change in the future. Our study suggests that a program of sustained corporate volunteering over several years may increase organizational learning and trust, leading to stronger capacity to advance and achieve NGO goals.

## Background

Sub-Saharan Africa is more heavily affected by HIV/AIDS than any other region in the world. More than 34 million HIV infected individuals live in East and Southern Africa alone [1]. Governments, non-governmental organizations (NGOs), citizen groups, and private industry all have an interest in stopping the spread of HIV in the region. Many are combining their efforts through partnerships designed to respond to the myriad challenges of research, service delivery, and policy advocacy [2,3]. Research partnerships face many challenges, including scarce research capacity, team work problems, logistical constraints, communication, and difficulties related to partner and community buy in and trust [4-7]. Effective partnerships therefore require investments in capacity building for international and local partners [8,9].

Capacity building is defined as “a process that improves the ability of a person, group, organization or system to meet its objectives or to perform better” [10]. Capacity building increases the ability and commitment to take effective action. Approaches to build capacity include financial and material support; organizational development activities to strengthen structures and institutional standard practices which enable smooth and effective running of operations or research projects; and building up of skills, confidence, and staff knowledge [5-7,11]. Capacity building activities are more successful when based on initial assessment of existing strengths and weaknesses; designed for sustainability over the long-term (e.g. training efforts also extend to stronger supervision, mentoring, and career development); and based on mutual respect and shared interests of the partners involved [4,5,9,11-13]. A key lesson learned from the literature is that capacity building is multifaceted, and neither cheap nor fast.

International volunteering is increasingly being used as a capacity building strategy [14,15]. Lough, et al. and others theorize that capacity building activities will lead organizations to greater performance and sustainability, often with reciprocal benefits for the volunteers themselves [15-17]. Benefits of capacity building in research include increases in successful grant applications, publications, dissemination activities, and influence of research findings on health policies and practice guidelines [11].

Yet, several factors make it hard to measure results in capacity building [10,18-20]. First, capacity is dynamic and continuous, so it cannot be easily measured using cross-sectional surveys. It is multidimensional, affecting variables at the system, organizational, and individual level. And capacity is very dependent on local context and the influence of the external environment [4,8]. In addition to these difficulties, it is not easy to determine the links between specific capacity building strategies and results. Changes in capacity may not proceed in a linear fashion and are shaped by interacting forces and actors [21]. Few studies to date have explored international volunteering as a capacity building strategy or attempted to show how it may help improve performance in the fight against HIV/AIDS.

To help fill this gap, we examined a partnership to promote capacity building through international corporate volunteering (ICV). This partnership brought employees from Pfizer Corporation, a multinational pharmaceutical company with over 116,000 employees, to work with the International AIDS Vaccine Institute (IAVI), an international NGO with 200 staff, to further the development of safe and effective vaccines to prevent AIDS. The case study describes how the Global Health Fellows (GHF) program helped develop capacity in IAVI and local partner organizations, looking specifically at eight Fellows who volunteered in South Africa, Kenya, and Uganda. After providing some background on the Pfizer GHF program and IAVI, the case describes capacity-building activities undertaken from 2005 to 2009, and the impact of those activities. While it is difficult to attribute changes to any single factor, our study suggests that a program of sustained volunteering may increase organizational learning, which in turn helps to strengthen organizational capacity for vaccine research. The findings also will suggest how contextual variables affect this process.

Pfizer Corporation’s GHF program is one of the first and most comprehensive ICV programs in the health sector [22-25]. Started in 2002, by 2010 over 250 Fellows had worked in 40 countries. Fellows are screened and selected to participate in the program through a competitive process at Pfizer. Partner organizations work with GHF staff to design the scope of work for the 3 to 6 month assignments and to choose a Fellow best matched to the assignment based on skills and experience. Pfizer then orients Fellows through a program which covers health, security, logistics, and cross-cultural management. During their assignment, the Fellow’s salary and benefits are paid from the budget of his or her work unit, while living allowance and travel costs are paid by the GHF program within Pfizer [23].

Started in 1999, IAVI works with partners in 25 countries and had an annual budget of nearly $84 million from 2005–2009. IAVI operates as a product development partnership (PDP), working closely with pharmaceutical companies, clinical research centers, and other partners to develop support for collaborative efforts to develop an AIDS vaccine ^a^. Its scientific teams, located on four continents, conduct research, develop products, and perform observational studies and sponsor clinical trials in partnership with more than 40 academic, commercial and government institutions. An important component of IAVI’s strategy is to conduct vaccine research and clinical trials in developing countries, particularly in East and Southern Africa. Table 1 summarizes the types of research studies and clinical trials which contribute to vaccine development.

**Table 1:** Descriptions of Research Studies and Clinical Trials

**Study**	**Description**
Observational study	A study where researchers are learning about HIV/AIDS without actually testing a drug or other intervention. Such a study might involve drawing blood from trial participants over time, to understand characteristics of non-infected individuals and to observe what happens at the cellular level when they become infected.
Pre-clinical trials	Studies of vaccines or treatment which are carried out in animals.
**Clinical trials**	
Phase I	Researchers test a vaccine in a small group of people (20–80) to determine how a drug should be given, how many doses are needed, and whether it is safe
Phase II	Experimental vaccine is given to a larger group of people (100–300) to see if it is effective and to further evaluate its safety
Phase III	Experimental vaccine is given to large groups of people (1,000-3,000) to confirm its effectiveness, monitor side effects, and compare to commonly used treatments.
Phase IV	Post-marketing studies determine a drug’s risks, benefits, and optimal use.

Source: http://clinicaltrials.gov/ct2/info/understand.

International standards for clinical trials are rigorous, and the same standards are applied whether participants are being enrolled in trials in Uganda, the United States, or any other country. This presents a challenge for vaccine research in low-resource settings where organizational capacity is often under-resourced. Lack of infrastructure for communications, shortages of trained personnel, and lack of community awareness are just some of the problems which research institutions in Africa must overcome in order to conduct vaccine trials. Efforts to address these problems are further challenged by the need to consider cultural values and community expectations related to vaccines.

Over a period of five years, from 2005–2009, 12 Pfizer GHF worked with IAVI and IAVI’s partners. Employees from Pfizer’s research divisions were selected for volunteer assignments with IAVI in South Africa, Uganda, and Kenya. The partnership between the two organizations had two goals: first, to help IAVI strengthen the capacity of its local partners to enable them to conduct vaccine trials meeting international standards; and secondly, to stimulate organizational learning within IAVI so the organization could continuously improve its ability to coordinate and manage an ever increasing portfolio of HIV/AIDS vaccine trials and other innovative research studies.

**Figure 1:**
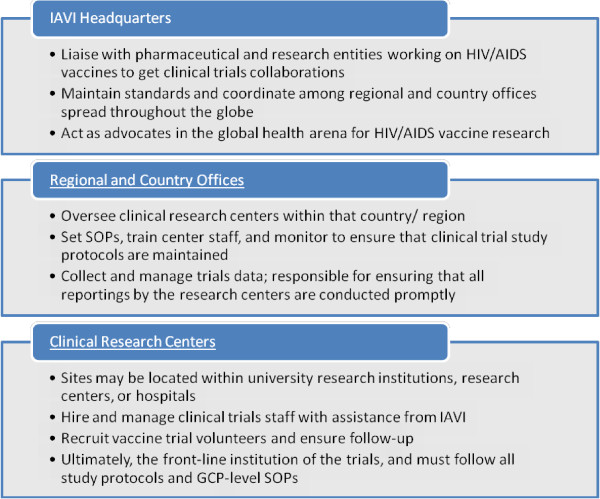
**IAVI Organizational Structure for HIV Vaccine Clinical Trials in Africa.** Source: Authors, adapted from IAVI web site content and journal entries by Pfizer Fellows.

This case study focuses on 8 fellowships during this period, each lasting 6 months. To select the fellowships we reviewed job descriptions to find projects focused mainly on clinical trial capacity building, and included only fellowships completed by 2009. Most Fellows worked at Regional and Country Offices or with the clinical trial centers in Uganda, Kenya, and South Africa, often administered by partner organizations (see Figure 1). In some cases, Fellows also traveled to other locations such as Zambia and Rwanda. Earlier Fellows worked mostly with the field centers (IAVI partners) to increase their capacity to perform international standard clinical trials, while more recent Fellows focused their efforts on how IAVI itself could strengthen capacity to manage multiple trials at once.

## Methods

Data was collected for this case study in two ways. First, we reviewed Pfizer administrative records of fellowships, including job descriptions, online journals (blogs) written by Fellows during their six-months in the field, final fellowship reports, annual individual evaluations of fellows by supervisors and the fellows themselves, and annual GHF program performance evaluations. In addition, follow-up interviews were held with Fellows and IAVI staff to probe for the long-term impact of GHF work and the potential benefit of sending multiple Fellows over several years – aspects which were never addressed in past individual and program-level evaluations.

We made multiple efforts to contact all 8 Fellows but were only able to interview 4: three had left the company and could not be located, and one was not available to interview within the data collection period. Although we could not conduct follow-up interviews with all Fellows, we had detailed evaluation information on all fellowships, often in the Fellow’s own words (e.g. through blog entries).

We also interviewed 5 staff identified by IAVI as having worked closely with individual Fellows and being well informed about their projects. The interviews asked Fellows and staff to describe IAVI’s level of development and capacity building needs at the start of the fellowships, GHF activities which seemed to have the most impact on capacity building, and perceptions about the structure, benefits, and challenges of the IAVI-GHF partnership over time. Interviews were conducted in August-September 2011. Our protocol did not include interviews with staff of research partner organizations or clinical centers; however, often the opinions of these staff were documented in fellowship evaluation reports or other administrative records.

## Results

### IAVI in 2005

In 2005, IAVI had limited research partners and clinical centers in Southern and East Africa, and was heavily reliant on contract research organizations for trial-related functions such as training and monitoring. While IAVI had been involved in several scientific and epidemiologic studies and had completed three HIV vaccine trials, the organization did not have a lot of experience managing multiple trials and trial centers at once. IAVI had established small field offices in Uganda and Kenya, and had one staff member working in South Africa. Often, trial management functions were carried out by IAVI staff operating from remote offices in New York, Netherlands and Belgium.

According to Fellows who volunteered in 2005 and 2006, IAVI was well organized and had a clear vision and mission. IAVI staff understood that in order to reach their goal of expanding HIV/AIDS clinical trial research in Africa, they needed to further develop the skills of African investigators, approach new partner organizations, and prepare research centers to meet the high standards required for clinical trials. Although IAVI staff had the expertise to conduct training and monitor multiple trials simultaneously, the organization did not have adequate resources to expand rapidly or to support the size of the trials envisioned. To ensure the timeliness of trial progress, the integrity of data, and the safety of trial participants, IAVI needed to ramp up training, and bring trial monitoring in-house.

Lean staffing of field offices also meant that IAVI did not have much back-up if anything went wrong. If a staff member with a professional skill set was sick or unavailable, there was “no one ready to step into that person’s shoes” without the need for extensive training. This situation is common to many non-profits, but in a time-sensitive and highly technical field such as clinical research, it is an especially important area of vulnerability.

### Contributions to capacity building

According to Lough, et al. (2011), key areas of direct contribution to capacity building by volunteers include: 1) promoting intercultural understanding; 2) providing or leveraging additional resources such as supplies or money; 3) extending service provision by acting as an “extra pair of hands”; and 4) applying technical and professional skills.

Long-term, skilled volunteers tend to provide added value in the latter two dimensions [14]; for example, by filling critical short-term staffing needs or by providing needed technical assistance, technology transfer, or staff development activities. Table 2 lists the 8 Fellows who worked in clinical trial capacity building and summarizes their job descriptions. The main ways in which Fellows applied their professional skills are described below.

**Table 2:** Capacity Building GHF Fellowships with IAVI

**Fellows**	**Country**	**Year**	**Position**	**Job responsibilities**
Fellow 1	South Africa	2005	Clinical Research Site Manager	Setting up study operations manual (SOM) and conducting center initiation activities and training for a 5-center Phase II trial in 3 countries
Fellow 2	Kenya	2005	Clinical Research Trainer	Setting up clinical research centers, conducting training on Good Clinical Practices (GCP)
Fellow 3	South Africa	2006	Sr. Clinical Research Associate	Developed SOMs and modules for GCP training, prepared centers for monitoring visits and audit
Fellow 4	Uganda	2006	Clinical Project Manager	Conducted monitoring, initiation of centers, and training of study personnel. Worked in Uganda and Zambia.
Fellow 5	Kenya	2007	Clinical Program Manager	Helped in center initiation activities including writing standard operating procedures, quality management plans, and preparing centers for inspection. Created training plans and trained investigators and trial participants
Fellow 6	Uganda	2008	Clinical Trial Site Monitor	Conducted GCP training, Clinical Research Associate (CRA) training. Developed monitoring tools and conducted monitoring visits with CRAs. Provided feedback and mentoring.
Fellow 7	Kenya	2009	Clinical Project Manager	Conducted GCP training, created monitoring tools
Fellow 8	South Africa	2009	Data Manager	Conducted a data management assessment, including documentation of current processes for lab sample movement, and other data flows. Made recommendations to IAVI board.

### Four areas of GHF technical assistance

Capacity building for clinical trials roughly can be divided into four phases which are mutually reinforcing (Table 3). First, during the start-up period the clinic centers must be identified and operating procedures established. These procedures need to be adapted for each particular research study which may involve different objectives, types of participants, safety concerns and data collection needs. Secondly, staff involved with current studies and possible future trials need to be trained so that they are aware of how to protect trial participants and comply with quality standards. Once the set-up period is over, a third set of activities involves building monitoring capacity (staff and systems) to assure that centers are audit-ready at all times and can demonstrate adherence to standards during regulatory visits. Finally, as the volume of clinical trials grows, the need to manage multiple clinical trials at once poses additional challenges. Data management systems need to be strong enough to organize, analyze, and allow access to data by many kinds of users in timely, efficient ways.

**Table 3:** Stages of Capacity Building for Clinical Trials

**Phase**	**Types of activities needed**
Start up	Assess center readiness, develop standard operations guides
Training	Train staff and others (as requested) on Good Clinical Practice (GCP)
Monitoring	Create supports and assure compliance with quality standards
Management	Design systems to manage multiple clinical trials at once

IAVI GHF Fellows provided technical assistance in these four areas: center preparation, training, monitoring, and data management. Although all areas included activities which benefited both IAVI and local partner organizations, the first two types of assistance tended to be focused on local partners, while the latter two were often targeted toward IAVI itself. Examples of the activities undertaken by Fellows in each area are described below.

### Start up and center preparation

Fellows were involved in diverse activities to prepare field centers to undertake specific studies and clinical trials. Capacity strengthening included writing standard operating procedures (SOPs) and study operations manuals (SOMs) adapted to research study protocol and the centers themselves; conducting center initiation meetings; and making on-site visits to review operations and procedures in clinics. These activities were generally undertaken by Fellows in 2005–2006, and sometimes involved centers which had not done many research studies, or which had never been involved in clinical research or HIV vaccine trials. Fellows often had to travel to other domestic and international locations; for example, one vaccine trial protocol involved three South African centers plus centers in Zambia and Uganda.

### Training

Fellows have provided assistance in training, with the topics evolving over time. At first, training focused on Good Clinical Practice (GCP). Developed by the International Conference on Harmonization (ICH), GCP is an international ethical and scientific quality standard for designing, conducting and reporting on trials involving human subjects. The GCP standard is meant to protect the rights and safety of participants in clinical research studies, and to help ensure scientific integrity and quality of findings. A basic understanding of GCP is prerequisite for anyone involved in clinical trials, including principal investigators, clinic support staff, monitoring personnel, ethics boards, and community oversight committees. In addition to receiving initial training, personnel should also receive refresher training on a regular basis.

Pfizer Fellows worked with IAVI staff to develop in-house training modules and conduct GCP training for center staff and others interested in receiving training (e.g. advisory board members) in Kenya, South Africa, Uganda, Rwanda and Zambia. In the past, IAVI had relied on contract organizations to conduct training, a strategy which ensured high quality but was expensive. A regional director explained how GHF Fellows helped IAVI change its training strategy and increase efficiency:

We used to outsource GCP trainings for the Africa network, but at a significant cost. The Fellows helped us to transition from this outsourced model to an in-sourced operation. We fully made the transition and no longer go outside for training resources. This has meant tremendous cost savings for IAVI.

Although IAVI still needs to update training curricula to keep it fresh, the role of IAVI local and central staff is growing more important. “Basically we’ve optimized the way we implement this required training,” said one IAVI manager. “We don’t need GHF assistance in this area anymore.” Another staff member concurred: “We now have African CRAs (Clinical Research Associates) with 3–5 years’ experience. They have had training, they’ve worked. They can do the job themselves.”

### Monitoring

Clinical studies require monitoring to protect participant safety and ensure integrity of data. Each monitoring plan must be adapted to context and depends on the study’s degree of risk, rate of enrollment of participants, experience of centers and principal investigators, etc. This presents a challenge for an organization like IAVI which is monitoring multiple trials and trial centers at once. Study managers and monitoring staff must make many decisions about what to monitor, how communications should be organized (including information shared by telephone, e-mail, or during monitoring visits), and how to handle deficiencies discovered during monitoring.

GHF Fellows worked with IAVI to develop monitoring plans and train staff. A focus of Fellows was to instill the principle that clinical research centers should be audit ready at all times, instead of reacting with panic once a regulatory visit is announced. Fellows helped IAVI to prepare plans which were responsive to different regulatory authorities and funders, including the US National Institutes for Health and others. They designed monitoring forms which would allow automated data input, to make it easier to track problems and compare centers. Some Fellows adapted training tools from Pfizer and from regulatory authority web sites. An IAVI regional director spoke about the result of GHF assistance in the Africa region:

In the last 6 months alone, we have vaccinated 300 people and we are monitoring it all ourselves, as opposed to using contracted monitors. So this is an example of how the capacity building has been not just for the local partners, but for IAVI Africa as well.

An additional challenge was to inculcate the belief that monitoring is important and that everyone’s participation is needed. Developing this type of commitment is important to support and sustain organizational capacity [26]. One Fellow explained:

Although the monitors had a good base of knowledge of GCP, they didn’t yet see the big picture. They knew the role and job description, but they didn’t realize how every little thing they have to review, every note they take, can make a difference. I tried to help them discover the true extent of the monitor’s responsibilities when it comes to overseeing the trials.

Teaching monitors how to give feedback is especially important in cultural settings where authority is centralized and power differentials may make it difficult for someone who is not the principal investigator to point out mistakes or give constructive criticism [5]. A Fellow recounted how she would mentor IAVI staff during training, to help build their confidence in working as a monitor in this context. “Your best argument is knowledge,” she would say. “If you aren’t certain, try to get the information.” She wanted the monitors to understand that giving feedback did not mean you were “attacking” the research team, but that it is part of the monitor’s job to “address the physician, to ask him to clarify. Not to let it go.” In a 2007 evaluation report, an IAVI supervisor described the impact of this mentoring [25]:

[The Fellow] introduced the idea that monitoring would start by looking at the last feedback letter, and making sure that those identified problems had been addressed. If they hadn’t, then the team would have a chance to address them while the monitor was present…It is very hard to give criticism in a way that causes action without [causing] resentment. But this is what the GHF was very good at, and we learned how the systems can support this.

### Data management

Investment in research infrastructure, including information systems, is another critical aspect of sustainable capacity building [11]. By 2009, IAVI’s investments in information technology were not keeping pace with its growth in clinical trial enrollment—and the data that accompanied this growth, especially data related to specimen tracking and association of research data with specimens. This made it hard for IAVI to take advantage of the data they had collected, as they lacked capacity to systematically collate, synthesize, conduct queries, and report on these data. To address these needs, IAVI created a job description for a Fellow to assess the data management function and make recommendations. The GHF Fellow selected for this position summarized the critical importance of data management for capacity building:

Data underlie everything in clinical trials. If you don’t fix the plumbing then you won’t get the water. Data flow, data exchange, access of data, analysis of data--people can only make changes once they know how things are unfolding, and they know this based on data. If they get the data at the right time, they can alter the way the clinical trial is happening.

The Fellow began by documenting how data were collected and flowed through the system based on field visits and interviews. The resulting process maps provided a more global view of how organizational units interacted and highlighted gaps and redundancies. IAVI staff found the maps useful: one manager said the process maps were referred to in new staff orientations, while another IAVI data manager described other benefits:

[Five months after the Fellow’s departure] we created a “sample management” task force which was trying to streamline and improve the way we managed laboratory samples. We began with the process map that [the Fellow] had done: it gave us a “coat hanger” of sorts, a place to start. We viewed each process and said ‘Ok, so this is how it works now. Where can we improve this?’ And we completely re-designed the process for sample tracking. The result is a simplified system that makes it much easier to track these samples in the database. It uses less forms and paper, less back and forth: it is a more efficient process now, used in all sites in Africa. And we started with [the Fellow]’s process maps.

The Fellow’s work also raised awareness of the need to take a broad and strategic view of data management activities. The IAVI data manager explained:

We thought we needed a data warehouse, and we decided that we were going to do it. But it didn’t fit into any longer term strategy, so, as the Fellow predicted, a year later nothing is happening. The Fellow really made that a point, the idea that we have to look at things overall, to take a broader view. We don’t have a strategy, so the initiative didn’t get traction.

Another IAVI staff member noted how the GHF had offered a longer-term and more strategic perspective: “That ‘view from-afar’ was very helpful. Sometimes at IAVI we don’t see the woods for the trees. The Fellow had this more objective view. Often the Fellow could provide simple solutions to problems that to us seemed very complex.”

Although the Fellow’s final report was presented to the IAVI senior management team, not all the recommendations could be implemented. For example, one important recommendation was the need to recruit someone to oversee all data management activities across the Research and Development function, but this was not done due to cost considerations and disagreement on how to integrate the new position into the organizational structure. At the same time, another IAVI director believes this Fellow’s work has really guided how IAVI collects data and adapts to change:

The senior management team is now aware of where we lack capacity, and since then we have employed additional people to manage data. The report opened everyone’s eyes to think about how we are collecting data, where we are putting data, and how we are sharing it. Things won’t change overnight, but people are much more aware of the issues now. Every time there’s something new to consider, we refer to those diagrams [of work process flow].

### Factors which promote effectiveness

We asked the Pfizer Fellows and IAVI supervisors to reflect on what factors in particular helped make their partnership and the international volunteering program more effective. We also analyzed Fellowship documentation to pinpoint variables which seemed to affect change. The factors identified included:

● similarities in mission between IAVI and Pfizer;

● the expertise, experience, and perspective of Fellows;

● openness;

● focus on organizational learning; and

● trust-building or motivation.

**Figure 2:**
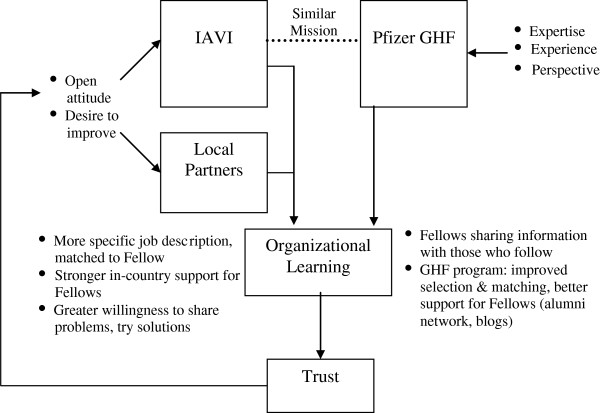
Model of Factors Predicting Success of ICV Partnership.

Figure 2 depicts the relationships among these factors.

### Similarities in mission

Similarities in the types of activities undertaken by Pfizer and IAVI staff, working to develop new medicinal products, allowed Fellows to be more effective. “We understood them and they understood us, because our missions and our backgrounds were similar,” said one Fellow. “We were able to start right away and to be helpful sooner, because the things they wanted us to do are things we do every day in our work.” Where GHF are assigned to work for organizations whose mission is not so similar, or where the job description requires a stretch, the learning curve can be greater. For example, a Fellow working with a humanitarian NGO in Northern Kenya was asked to create a database to manage refugee information. While the Fellow had strong skills in database management, it took her some time to get to know the organization and the issues. The learning curve for meeting the challenges of clinical trial management was shorter for Pfizer Fellows assigned to IAVI.

### Expertise, experience, and perspective of fellows

A second factor mentioned by many informants was the expertise, experience, and broad perspective of Fellows. Non-profits like IAVI are stretched and may not always have expertise available at the time and place it is needed. One way to cover these gaps is to recruit a Fellow who can quickly contribute scarce skills or knowledge. As one Fellow remarked, “Pfizer has people like me in the thousands, but small NGOs do not. At the same time, the NGOs are nimble and agile institutions: this allows them to use our expertise well when they have it.” Another way in which Pfizer Fellows’ expertise has been tapped is when a Fellow steps into a job which is temporarily vacant. An IAVI supervisor described how this happened in South Africa:

The timing couldn’t have been better, because as soon as she came, IAVI’s senior Clinical Research Associate (CRA) fell ill. The Fellow was able to take over for six months, just like nothing had happened. She had a big and fast learning curve, but she was so senior that she really knew what she was doing, she knew exactly what needed to happen. Without the Fellow, we would have fallen behind in the protocol; the study would have been delayed.

Working in South Africa, the Fellow compiled an SOM for a new trial and helped two centers to begin recruitment of trial participants. She trained staff on GCP and helped them prepare for audit and inspections. The Fellow described the advantage of using highly experienced volunteers, “We can hit the ground running, doing things that, if IAVI had to hire staff, they would have had to train people to do… None of us are novices. Having that experience is very helpful for IAVI.”

IAVI staff also mentioned how experience, maturity, and being able to handle uncertainty make Fellows more productive. IAVI has learned to be cautious about accepting an otherwise technically-qualified Fellow who doesn’t have relevant experience living or working in a developing country. “If a Fellow has never traveled, never come to Africa, then we will have reservations about them going to a rural site,” said one supervisor.

Finally, IAVI staff noted that the Fellows’ leadership skill was important to their effectiveness, i.e., their ability to bring a broader perspective, scanning the environment beyond the work plan. Pfizer Fellows helped IAVI step back from the day-to-day “fire-fighting” and think about strategic steps needed to realize IAVI’s mission. As one Fellow explained, “[Pfizer employees] are used to looking for issues, anticipating, scanning the situation. We are used to identifying issues before they occur. IAVI wasn’t used to looking ahead, in part because they just didn’t have enough people. So we could help them do that.”

### Open attitude and desire to improve

Literature suggests that organizational readiness for capacity building enables better results. Organizational readiness includes organizational willingness to question their current practices and commitment to participate actively in capacity building activities [9]. A third factor which was important to the effectiveness of the Pfizer-IAVI partnership was an open attitude and desire to improve on the part of IAVI and the local partner organizations. Fellows noted IAVI’s interest in receiving advice to improve their operations, their “teamwork, spirit of motivation, supportive and sympathetic principal investigators” at the clinic centers. “IAVI staff took feedback well and acted on recommendations thoughtfully,” said one Fellow. Another Fellow noted how IAVI’s interest in improvement extended to interactions with partner organizations: “IAVI would listen to ideas for how a change in processes could help their partner organizations. They knew they could only accomplish things if the partner organizations are effective, so these kinds of changes helped IAVI too.”

Even the most qualified and experienced international volunteers cannot build capacity if the environment is not receptive. According to GHF Fellows, the effectiveness of their efforts depended on the caliber of African staff at the research centers. Fellows worked with highly motivated staff at these clinic centers, carefully selected people who really wanted to make a difference. They were trying to work with the local community. There was a huge amount of education needed in the community and many misconceptions about HIV/AIDS. So the staff commitment was important.

### Focus on organizational learning

Both Pfizer and IAVI learned from the experience of early Fellows, making changes to improve support of Fellows. IAVI tried to improve their readiness to receive volunteers in many ways. They arranged safe accommodations ahead of time, increased budgets to be sure that Fellows would have easy mobility, and engaged their own staff in orienting and working with the Fellows. IAVI staff made efforts to seek feedback from Fellows on how they could improve field program design and overall organization. Over time, these changes allowed Fellows to provide substantive contributions earlier in the fellowship period. One IAVI supervisor explains:

The fact that Johannesburg office was only established in 2006 has bearing on the support we were able to provide for the first Fellow when she came in 2005. It was a challenge for her to be in place without an established IAVI office. We hadn’t thought through how to handle the fellowships. IAVI learned a lot of lessons and changed things so that subsequent Fellows could be more effective.

Later Fellows confirmed that these improvements eased their transition. For example, although it took early Fellows several days to get an apartment and a cell phone, later Fellows found that IAVI had arranged suitable living space and met them at the airport with a cell phone pre-programmed with staff contacts.

Another area of organizational learning was in defining job descriptions, Fellow selection, and matching. IAVI staff said they learned to make the job descriptions very clear, and to be sure that the tasks were feasible to accomplish in the time allowed. “Now we create defined deliverables,” said one supervisor. “We want to make sure that the Fellows are aware of what we expect.” Both IAVI and Pfizer GHF program staff recognize that having a good match between the Fellow and the host organization is a critical success factor. One Fellow explained, “The organization’s need has to match with the talent from Pfizer. This is the most important thing. If they don’t have the right person to solve the problem, there will be disappointment on both sides.” Another Fellow agreed, stating that NGOs need to take the time to make the right match between what a particular Fellow knows how to do, and the kind of help the organization needs. In this way they can “tweak” the assignment to take best advantage of the Fellow’s help: “When ‘match’ meets ‘talent and passion’, things turn out right.”

GHF program staff and individual Fellows also took actions to improve the program based on past experience. For example, earlier Fellows made themselves available to meet with Fellows just going out, to share experiences and help the new Fellows understand the institutions, locations, and environment. One Fellow explained, “A Fellow who was going to this location after me was very afraid of the security issues. I spoke at length to her, to give her practical suggestions and to reassure her.”

Fellows held debriefings with IAVI at the end of their stay, to discuss lessons learned which might affect the design of the next Fellow’s job description. In such meetings, the Fellow and IAVI staff discussed positives and negatives of the fellowship, what could be done better, and how IAVI and GHF program could make changes to further maximize the capacity building contributions of Fellows. “Part of what is important to this is that the Fellows talk with IAVI, at the start and throughout,” observed one Fellow. “We have to relate our experiences to their situation and their plans. We talk about what we have done. You keep enlarging the picture.”

Pfizer GHF program staff also tried to improve communication channels and facilitate learning. The program added guidance to help partner organizations design more specific job descriptions, helped Fellows to blog about their experiences, and created a GHF alumni network to promote information sharing.

In designing job descriptions for Fellows, IAVI staff often sit down together as a team. Sometimes they can design fellowships to build on one another, but this is not easy due to timing and the candidates available. Yet, one Fellow noted that even if each Fellow has somewhat different skills or experience and works on a different problem, they are contributing something toward the overall goal of capacity building: “It is a bit like a jigsaw puzzle. We each put a little into it, and it makes the big picture.”

### Trust

Trust is the basis of any stakeholder collaboration and enhances capacity building [3,6,9,12]. The organization receiving a Fellow needs to believe that the Fellow has the organization’s interests in mind, and has something of value to offer. Trust is also a quality that is personal. Just because an organization has had several Fellows, does not mean they will trust every Fellow. It is people working together during a fellowship who generate trust. As one Fellow noted, “You have to go beyond their duties to get to know the people. Only then will they accept what you are saying.”

While the focus of this case study is the impact of corporate volunteering in building NGO capacity, the Fellows who participated in the program also gained skills and experience working in new regions or a new therapeutic area. Fellows learn to adapt to challenges such as lack of electricity, travel mishaps, and a “different beat” in the work place--logistical constraints which are a major challenge for researchers in low-income settings [4]. For example, one Fellow stated that “I learned that I needed to start earlier to get things done, and to accept that some things were going to happen last minute.” Another area of learning was about culture differences. “You can ask questions about sexual behavior in Kenya that you can’t ask in Uganda,” said an IAVI regional director. “Everywhere we work the cultures are different. The best Fellows see this as an opportunity to learn.” A Fellow who worked in South Africa described how her fellowship helped her appreciate how cultural values influence clinical trials:

The concept of informed consent is that the patient can withdraw at any time. Yet, when I was talking to colleagues in East Africa, they didn’t see it like that. They thought, ‘If we are spending this amount of money, we want these patients to continue. Why are you telling them they can withdraw? That gives the impression you are not serious about what you are doing.’ Once you understand these cultural differences, you learn how to explain things in a different way, so they can see why you are insisting on doing this strange thing that seems wrong to them. Nowadays, pharmaceutical companies are conducting trials all over the world, in places like India, China. I have not been to those countries, but if I am asked to go, I can imagine that similar sorts of cultural issues will be important.

## Discussion

Developing capacity leads to greater ability to perform useful research [11]. The partnership between IAVI and Pfizer was successful in building capacity for clinical trials in the areas of center preparation, training, monitoring, and data management systems. Fellows helped IAVI and local partner staff to develop clinical trial procedures, conduct GCP training, and design monitoring systems to assure that trial centers are audit ready at all times. They helped IAVI in periods of growth and transition: expanding from doing small, sequential trials to running large trials simultaneously in multiple centers and countries; transitioning from contracted out training and monitoring services to using less expensive but equally high quality in-house capacity; anticipating data management needs and streamlining processes to share information more efficiently and effectively.

The successes in the partnership can be traced to multiple factors, including similarities in mission between IAVI and Pfizer, which meant that Fellows needed less time to become productive; the high levels of expertise and experience which Fellows could draw upon, and the broad perspective they could offer to IAVI as they helped the NGO to improve or expand into areas of opportunity; attitude and motivation of IAVI and the local partners, who were open and willing to work on problems and try new approaches; and an orientation toward organizational learning on the part of IAVI and Pfizer’s GHF program, which allowed both organizations to adapt and improve the partnership based on past experience.

Over the five years of collaboration, these factors and the positive results achieved built greater trust which in turn reinforced the model. As Fellows and IAVI staff and partners had positive experiences working together, and as IAVI achieved meaningful improvements in their capacity to achieve their goals, the partners were more willing to experiment, work harder, and test ways to improve the program even more.

Some elements of the Pfizer-IAVI partnership’s success reflect factors identified in the literature on capacity building; for example, IAVI’s readiness for capacity building, the long-term nature of the partnership which allowed in-depth understanding of needs and time to develop capacity, and the adaptation of capacity building strategies to the specific needs of vaccine research.

Another influence on the success of the IAVI-Pfizer collaboration is how IAVI thought about the assistance. The drive to use corporate volunteer assistance seemed to come not from any specific gap that IAVI was experiencing, but from the desire to learn; i.e., ‘What do we want to know from industry?’ , ‘How can GHF be a catalyst for capacity building in this context?’ Given this motivation, capacity building assistance was not perceived as part of a linear plan but rather as a mosaic: a mechanism for “opportunistic improvement” guided by the organizational mission and the experience, skills and perspective of the Fellows themselves. The importance of this type of ‘organizational learning’ orientation and its link to capacity building has not been previously noted in the literature. Health professionals designing public-private partnerships in other contexts may want to consider approaches to deliberately promote an organizational learning culture and take advantage of the fertile environment provided for development.

All this is not to say that the partnership did not experience bumps and disappointments along the way. Some of the challenges described by the participants included lack of follow-up after a fellowship, an individual Fellow who didn’t work out, and staffing turnover at IAVI.

Several Fellows and IAVI staff mentioned the difficulty in arranging for sequential Fellowships that would build on each other. “It would have been helpful if someone came right after me, to implement the recommendations I helped generate,” said one Fellow. Yet, sequential fellowships are hard to arrange. “We haven’t always had a lot of people to choose from” said an IAVI regional director, “because often the job description is very specific, and finding a match is not guaranteed.” The GHF program has been re-designed in recent years to encourage more sequential fellowships, a strategy which may help address this problem. As an alternative to arranging sequential fellowships, the program might consider longer fellowships or extensions as a way of ensuring that important projects are completed.

IAVI has had very good experience to date in selection of Fellows; however, in one case a Fellow did not achieve the results expected. To minimize unproductive fellowships, Pfizer has tried to strengthen screening of candidates and helps recipient organizations to develop clear and specific job descriptions. IAVI has also taken action to increase internal participation in the selection process and to interview candidates more carefully. Although IAVI managers thought having the occasional bad match may be unavoidable, one Fellow thought it was important to design a system for early termination of fellowships that don’t seem to be working: “There should be a way to terminate the relationship, so as not to waste everyone’s time and make others discouraged who hear about the waste.”

Finally, inadequate staffing in the organization receiving the Fellow can make it difficult to achieve results. One Fellow noted that the South Africa regional office was currently operating with one-third the staff it had in 2009. What this means is that people are busy getting their work plan done, and don’t have time to “step out of the box, to take time to think”. This can be an impediment to organizational learning.

### Limitations

It is clear that the issue of capacity building and PDPs is much broader than the remit of this article [27]. The focus of this study is the capacity building contribution made by Pfizer Fellows to IAVI rather than the contribution made by IAVI to the developing countries in which it is active. The latter topic is written about in papers by Chataway and Hanlin [28] and others [29].

Our study was limited by several factors. As mentioned earlier, we were only able to re-interview four of the eight Fellows, though we did analyze archival material (e.g. job descriptions, online journal entries, prior evaluation reports) on each of the eight fellowships. In addition, we limited the study to Fellows whose job descriptions were directly related to clinical research capacity building, thus excluding other Fellows whose work indirectly supported capacity building (for example, several IAVI staff mentioned a Fellow who mentored laboratory staff in Flow Cytometry, including one laboratorian who is now viewed as a regional resource because of his expertise). Finally, given that our purpose was to characterize the relationship between the international Fellows and IAVI as an organization, we conducted interviews with Fellows and IAVI staff. However, Fellows also worked with local Clinical Research Centers to build capacity in various areas. Perspectives of key CRC staff such as directors and Principal Investigators who interacted with the Fellows could have provided further information on capacity strengthening for these IAVI partners. This would be an interesting topic for further study.

## Conclusion

International corporate volunteering programs like the Pfizer Global Health Fellows program can help build stronger capacity for international and local NGO partners. By developing standard operating procedures, ensuring that monitoring and regulatory compliance systems were in place, training African investigators and community members, and engaging in other systems strengthening activities, GHF has helped IAVI to accelerate vaccine development activities in the field while also developing the organization’s capacity to manage change in the future. Corporations and donors working in public-private partnerships to further public health goals may wish to consider adding an international volunteering component as a capacity strengthening strategy to complement other efforts. More work is needed to document these types of programs and their impact over time.

## Endnote

^a^An AIDS vaccine is defined as “an experimental strategy that aims to teach the body’s immune system how to fight HIV to reduce the risk of infection or to reduce viral load in those who get the vaccine and go on to become infected.” http://www.avac.org/ht/d/sp/i/324/pid/324 Accessed 9/20/11.

## Competing interests

Boston University Center for Global Health and Development (CGHD), the employer of the authors at the time of the study, has received funding from Pfizer Corporation’s Social Responsibility division to evaluate the Global Health Fellows program. The creation of this case study is part of the evaluation work conducted under this contract. The contract also will fund article processing charges.

## Authors’ contributions

JB, TV and FF are currently on the faculty of the Department of International Health and researchers at CGHD. They were involved in conceptualizing the study and design of methods. TV carried out interviews, analyzed administrative records, transcripts, and other data, and drafted the manuscript. SK was a Research Assistant at CGHD at the time of the study. She made substantial contributions to the design of the study, identified and contacted key informants, collected administrative data, and participated in the drafting of the manuscript. All authors reviewed and commented on earlier drafts and approved the final manuscript.

## Pre-publication history

The pre-publication history for this paper can be accessed here:

http://www.biomedcentral.com/1472-6963/13/378/prepub
